# Factors that Affect the Surveillance and Late-Stage Detection of a Newly Diagnosed Hepatocellular Carcinoma

**DOI:** 10.31557/APJCP.2021.22.10.3293

**Published:** 2021-10

**Authors:** Attapon Rattanasupar, Supattra Chartleeraha, Keerati Akarapatima, Arunchai Chang

**Affiliations:** 1 *Division of Gastroenterology, Department of Internal Medicine, Hatyai Hospital, Songkhla, Thailand. *; 2 *Department of Internal Medicine, Hatyai Hospital, Songkhla, Thailand. *

**Keywords:** Hepatocellular carcinoma, hepatitis B virus, liver neoplasm, Thailand

## Abstract

**Background:**

Surveillance of hepatocellular carcinoma (HCC) is beneficial for detecting early-stage HCC. The factors that influence adherence to HCC surveillance and late-stage detection have never been evaluated. We investigated the predictive factors that contribute to patients accessing regular HCC surveillance and their association with the detection of late-stage HCC at the time of diagnosis.

**Methods:**

We conducted a prospective observational study at Hatyai Hospital (Songkhla, Thailand) between 2014 and 2016. HCC surveillance includes performing hepatic ultrasonography with/without serum alpha-fetoprotein 6–12 months before the detection of HCC. Logistic regression analyses were conducted separately to examine the relationship between the variables and each endpoint.

**Results:**

One hundred ninety-nine HCC patients were enrolled in the study; most patients were of low socioeconomic status, 90.5% had less than a bachelor’s degree, and 69.3% of patients had a monthly income of <10,000 baths (US $312.50). Nearly all (93.5%) patients had cirrhosis, 39.7% had hepatitis B virus (HBV) infection, 24.6% had hepatitis C virus infection, and 24.6% had alcohol-related liver disease. The risk of HCC was recognized in 51.8% of patients, and regular HCC surveillance was achieved in 36.2% of patients. Multivariate logistic regression analysis revealed that a monthly income >10,000 baths (US $312.50) (odds ratio [OR], 4.566; p = 0.013), HBV infection (OR, 0.188; p = 0.001), and recognition of patients at risk of HCC (OR, 130.396; p<0.001) were independent predictive factors for adherence to HCC surveillance. Regular HCC surveillance (OR, 0.215; p = 0.003) and recognition of HBV infection (OR, 0.356; p = 0.040) were independent preventive factors for the detection of late-stage HCC at the time of diagnosis.

**Conclusion:**

In Thailand, awareness of patients at risk of developing HCC and the rate of regular HCC surveillance are low. Greater awareness will enable physicians to surveil and detect HCC.

## Introduction

Hepatocellular carcinoma (HCC) is the second leading cause of cancer-related mortality worldwide (Taskaeva et al. 2018, Torre et al., 2015). In Asian countries (including Thailand), an endemic area of chronic hepatitis B virus (HBV) infection, the HCC incidence increases with advancing age (Massarweh and El-Serag, 2017). For the early stage of disease, curative therapy is recommended. However, more than one-half of patients with HCC are diagnosed when the disease is in the advanced stage, resulting in a tendency toward palliative treatment (Ahmad et al., (2020); Aljumah et al., 2016; Chang et al. 2018; Xiang et al., 2017). Current international guidelines, including guidelines of the Association for the Study of Liver Disease (AASLD) and the European Association for the Study of the Liver, recommend HCC surveillance using ultrasound (US) with or without serum alpha-fetoprotein (AFP) measurements in patients at high risk of HCC (European Association for the Study of the Liver, 2018; Marrero et al., 2018).

HCC surveillance (using regular hepatic US with/without AFP) increases the rate of detecting early-stage HCC, thereby resulting in patients receiving curative treatment and possibly improving survival outcomes (Im et al., 2019; Stravitz et al., 2008; Yang et al., 2011). Despite these well-known beneficial effects, the adoption of HCC surveillance in clinical practice remains suboptimal and accounts for 20%–70%; this varies among countries (Francica and Borzio, 2019; Singal et al., 2012). In Thailand, the rate of HCC surveillance adherence is 20% (Chaiteerakij et al., 2017). Previous data from central and northern Thailand show that more than 80% of HCC patients were in the late stage of disease at the time of diagnosis (Leerapun et al., 2013; Somboon et al., 2014), which could be explained by the low rate of HCC surveillance.

However, data on factors that influence adherence to HCC surveillance and late-stage detection have never been evaluated. Thus, the purpose of this study was to investigate predictive factors contributing to patients accessing regular HCC surveillance and the association of these factors with the detection of late-stage HCC at the time of diagnosis.

## Materials and Methods

*Study population*

This prospective cross-sectional study was conducted between September 2014 and December 2016. The study protocol was approved by the Ethics Committee of Hatyai Hospital Institutional Review Board and was conducted based on the Declaration of Helsinki guidelines. Informed consent was obtained from all participants. We enrolled HCC patients aged ≥18 years at the Hepatocellular Carcinoma Clinic, which was developed to register newly diagnosed HCC patients and provide them access to suitable treatment by a multidisciplinary team, in Hatyai Hospital, Songkhla, Thailand. The exclusion criteria included patients who had a previous diagnosis of HCC or who had previously received specific treatment for HCC at other hospitals. Patients’ information, including demographics and socioeconomic data, adherence to surveillance, etiology of baseline liver disease, and tumor stage, was obtained at the time of diagnosis.

*Diagnosis and definition*

A diagnosis of HCC was determined, based on the guidelines of the American Association for the Study of Liver Disease (AASLD) and/or the European Association for the Study of the Liver (Marrero et al., 2018; European Association for the Study of the Liver, 2018). Liver lesions larger than 1 cm in patients with cirrhosis or chronic HBV infection were diagnosed, based on dynamic cross-sectional imaging techniques (e.g., multiphasic computed tomography [CT] and dynamic contrast-enhanced magnetic resonance imaging [MRI]). Typical HCC was defined as early enhancement in the arterial phase and rapid washout in the venous or delayed phase and required no further investigation. A pathological diagnosis was obtained in ambiguous cases. The tumor stage was classified using the Barcelona-Clinic Liver Cancer (BCLC) classification (Marrero et al., 2018) into “early-stage” (i.e., BCLC stages 0 and A) or “late-stage” (i.e., BCLC stages B, C, and D).

Cirrhosis was diagnosed, based on clinical features, imaging, and/or histology. Patients at high risk of HCC had one of the following conditions: (1) cirrhosis from any cause, (2) chronic HBV infection and a history of HCC in first-degree relatives of any age, (3) chronic HBV infection in men ≥40 years old or in women ≥50 years old. Adherence to HCC surveillance was defined as hepatic US examination with or without serum AFP assessment conducted at least 6–12 months before the first detection of HCC. Recognition of patients at risk of HCC was determined if patients acknowledged that they were at high risk of developing HCC before receiving a diagnosis of HCC. Alcohol-related liver disease was considered at the alcohol consumption threshold of at least 40 g daily for 5 years (Grant et al., 1988).

*Statistical analysis*

Continuous variables are presented as the mean (standard deviation). Categorical variables are presented as frequencies and percentages. We aimed to identify independent affecting factors of “adherence to HCC surveillance” and “late-stage HCC at first diagnosis”. We measured both outcomes during the first HCC diagnosis according to the definition described above. Logistic regression analyses were conducted separately to examine the relationship between each outcome and the clinical and socioeconomic factors using the following variables: sex, age, income, highest education level, health insurance, cirrhosis, etiology of chronic liver disease (e.g., HBV, hepatitis C virus [HCV], alcohol-related liver disease), and recognition of being at risk of developing HCC. After conducting univariate analysis, age, sex, and other variables with a p-value (probability) of < 0.1 were included in the multivariate analyses. All analyses were conducted using Stata (version 15.1; StataCorp, LLC, College Station, TX). A value of p < 0.05 was statistically significant.

## Results

*Patient population*

A total of 199 newly diagnosed HCC patients were enrolled in the study. One hundred forty-one (70.9%) patients were men and the patients’ mean age was 58.6 ± 11.3 years. The patients’ demographic data are summarized in Table 1. Most patients were classified as having a low socioeconomic status, 180 (90.5%) patients graduated with less than a bachelor’s degree, and 138 (69.3%) patients had a monthly income of <10,000 baths (US $312.50). One hundred fifty-five (77.9%) patients used the National Universal Coverage Scheme. Based on the BCLC staging system, 17 (8.5%) patients were classified as having stage 0; 27 (13.6%) patients, stage A; 63 (31.7%) patients, stage B; 59 (29.6%) patients, stage C; and 33 (16.6%) patients, stage D. Early-stage HCC and late-stage HCC were determined in 22.1% and 77.9% of patients, respectively. Most (93.5%) patients had cirrhosis. The etiologies of baseline chronic liver disease were HBV, HCV, and alcoholic liver disease in 79 (39.7%), 49 (24.6%), and 49 (24.6%) of patients, respectively. However, only 51.8% of patients recognized that they were at risk of developing HCC, and 36.2% of patients underwent regular HCC surveillance. Of 79 patients with HBV infection, only 27 (34.2%) patients were aware of having the disease, whereas 86 (46.2%) of 186 cirrhotic patients recognized that they had cirrhosis before their diagnosis of HCC.

*Predictors of adherence to HCC surveillance*

To identify the predictive factors for regular HCC surveillance, a logistic regression analysis was conducted. In univariate analysis, the prognostic factors of regular HCC surveillance were a monthly income of >10,000 baths (US $312.50), having graduated with less than a bachelor’s degree, HBV infection, and recognition of patients at risk of HCC. Multivariate analysis revealed only a monthly income of >10,000 baths (US $312.50) (odds ratio [OR], 4.566; 95% confidence interval [CI], 1.370–15.215, p = 0.013), HBV infection (OR, 0.188; 95% CI, 0.073–0.486, p = 0.001), and recognition of patients at risk of HCC (OR, 130.396; 95% CI, 32.270–526.902, p<0.001) were independent predictive factors for adherence to HCC surveillance (Table 2).

*Predictors of the detection of late-stage HCC at first diagnosis*

Univariate analysis revealed that adherence to HCC surveillance and the recognition of patients at risk of HCC were predictive factors for the detection of late-stage HCC at first diagnosis. However, as described earlier, the association between adherence to HCC surveillance and recognition of patients at risk of HCC was extremely high. An explanation for this situation may be that most patients who recognized that they were at high risk of developing HCC adhered to HCC surveillance. For this reason, the recognition of patients at high risk of HCC was not included in the multivariate analysis and was subclassified into two groups (i.e., recognition of HBV infection and recognition of cirrhosis) for recalculating the multivariate logistic regression analysis. Multivariate analysis revealed that only regular HCC surveillance (OR, 0.215; 95% CI, 0.079–0.583, p = 0.003) and recognition of HBV infection (OR, 0.356; 95% CI, 0.133–0.953, p = 0.040) were independent preventive factors for the detection of late-stage HCC at the time of diagnosis (Table 3).

**Table 1:** Demographic Data

Variables	All (n=199)
Age (years): mean ± SD	58.6 ± 11.3
Female sex	58 (29.1%)
Health insurance	
National Universal Coverage Scheme	155 (77.9%)
Social Security Scheme	22 (11.1%)
Government Officer	20 (10.1%)
Self-payment	2 (1.0%)
Income (bath/month)	
less than 10,000	138 (69.3%)
10,000-30,000	53 (26.6%)
30,000-50,000	6 (3.0%)
More than 50,000	2 (1.0%)
Level of education	
Under Bachelor’s degree	180 (90.5%)
Bachelor’s degree or higher	19 (9.5%)
Baseline cirrhosis	186 (93.5%)
Etiology of chronic liver disease	
HBV infection	79 (39.7%)
HCV infection	49 (24.6%)
Alcohol-related liver disease	49 (24.6%)
Recognition of at-risk patients for HCC	103 (51.8%)
Recognition of cirrhosis	86 (43.2%)
Recognition of HBV	27 (13.6%)
Recognition of HBV cirrhosis	10 (5.0%)
Adherence to surveillance for HCC	72 (36.2%)
Barcelona Clinical Liver Cancer staging	
Stage 0	17 (8.5%)
Stage A	27 (13.6%)
Stage B	63 (31.7%)
Stage C	59 (29.6%)
Stage D	33 (16.6%)

The data are expressed as the number (%), unless otherwise specified; SD, Standard deviation; HBV, Hepatitis B virus; HCV, Hepatitis C virus; HCC, Hepatocellular carcinoma

**Table 2:** Factors Associated with the Adherence to HCC Surveillance in Patients Diagnosed with Hepatocellular Carcinoma

	Univariate analysis			Multivariate analysis		
Variables	OR	95%CI	P	OR	95%CI	P
Age, every 1-year increase	1.016	0.990-1.042	0.236	1.038	0.994-1.084	0.088
Female sex	1.368	0.730-2.563	0.328	1.306	0.469-3.640	0.609
National Universal Coverage Scheme	0.537	0.272-1.060	0.073	0.996	0.323-3.075	0.995
Income more than 10,000 bath/month	2.445	1.314-4.548	0.005	4.566	1.370-15.215	0.013
Education of under Bachelor’s degree	0.292	0.109-0.779	0.014	1.449	0.295-7.110	0.648
Cirrhosis	10,203.000	0-∞	0.999			
Alcohol-related liver disease	1.300	0.671-2.519	0.437			
HBV infection	0.540	0.293-0.996	0.048	0.188	0.073-0.486	0.001
HCV infection	1.456	0.753-2.814	0.264			
Recognition of at-risk patients for HCC	65.515	19.302-22.370	<0.001	130.396	32.270-526.902	<0.001

OR, Odds ratio; CI, Confident interval; HBV, Hepatitis B virus; HCV, Hepatitis C virus; HCC, Hepatocellular carcinoma

**Table 3:** Factors Associated with Late-Stage HCC at the Time of Diagnosis, Based on BCLC Staging

	Univariate analysis			Multivariate analysis		
Variables	OR	95%CI	P	OR	95%CI	P
Age, every 1-year increase	0.999	0.970-1.029	0.946	1.01	0.973-1.048	0.605
Female sex	0.65	0.320-1.322	0.234	0.611	0.267-1.399	0.244
National Universal Coverage Scheme	0.882	0.387-2.009	0.764			
Income more than 10,000 bath/month	0.816	0.400-1.663	0.576			
Education of under Bachelor’s degree	1.725	0.615-4.838	0.3			
Adherence to HCC surveillance	0.151	0.072-0.316	<0.001	0.215	0.079-0.583	0.003
Cirrhosis	0.277	0.035-2.192	0.224			
Alcohol-related liver disease	1.143	0.517-2.526	0.741			
HBV infection	1.198	0.599-2.396	0.609			
HCV infection	0.836	0.391-1.787	0.644			
Recognition of at-risk patients for HCC	0.230	0.106-0.498	<0.001	N/A	N/A	N/A
Recognition of HBV	0.419	0.176-0.996	0.049	0.356	0.133-0.953	0.04
Recognition of cirrhosis	0.231	0.112-0.477	<0.001	0.566	0.201-1.595	0.566

BCLC, Barcelona-Clinic Liver Cancer; OR, Odds ratio; CI, Confident interval; HBV, Hepatitis B virus; HCV, Hepatitis C virus; HCC, Hepatocellular carcinoma

## Discussion

In this study, we investigated the predictive factors contributing to patients accessing regular HCC surveillance and their association with the detection of late-stage HCC at the time of diagnosis. The main findings of our study were as follows. First, one-half of newly diagnosed patients were aware of their diagnosis of being at risk of HCC, and only one-third adhered to regular HCC surveillance. Second, a monthly income of >10,000 baths (US $312.50) (OR = 4.566) and recognition of patients at risk of HCC were favorable factors (OR = 130.396). By contrast, HBV infection (OR = 0.188) was an unfavorable factor for adherence to HCC surveillance. Third, adherence to HCC surveillance (OR = 0.215) and recognition of HBV infection (OR = 0.356) were preventive factors for the detection of late-stage HCC at the time of diagnosis.

The beneficial effects of HCC surveillance have been established in early detection, in enhancing patients receiving curative therapies, and possibly in improving overall survival (Ahmad et al., (2020); Im et al., 2019; Stravitz et al., 2008; Yang et al., 2011). A major step for ameliorating the adherence to HCC is identifying which patients are at risk of developing HCC. Unawareness of having baseline chronic liver diseases, such as cirrhosis or HBV infection, ranged from 23%–50%, based on the findings of studies in western countries (Ladhani et al., 2020; Shah et al., 2015). Consistent with our study’s findings, one-half of the patients with HCC realized that they had a disease with cancer risk. This finding underscores that the underdiagnosis of the risk of developing HCC remains a critical problem worldwide.

Most patients with HCC were diagnosed at the late stage of the disease. In this study, our data showed early-stage detection of newly diagnosed HCC accounting for 22% of diagnoses; this was comparable to the findings of previous studies in Thailand (Leerapun et al., 2013; Somboon et al., 2014), which revealed a rate of early diagnosis of 13%–16%. Furthermore, the rate of regular surveillance of HCC in the present study (36.2%) corresponded to the rate reported in recent meta-analyses, which reported that HCC surveillance was put into real-world practice in only 24%–37% of patients (Fernandes et al., 2020; Zhao et al., 2020). Underuse of HCC surveillance varies among different regions globally, depending on various factors, such as regional culture, environment, personal beliefs, and socioeconomic status (Dai et al., 2020; Dalton-Fitzgerald et al., 2015; Dirchwolf et al., 2021; Farvardin et al., 2017). Financial issues and the type of insurance system influence the assessment of HCC surveillance in western countries (Dalton-Fitzgerald et al., 2015; Farvardin et al., 2017). However, data from national health systems in China revealed that socioeconomic status had a minimal effect on assessing HCC surveillance (Dai et al., 2020). In Thailand, all health insurances (including the National Universal Coverage Scheme, Social Security Scheme, and Government Officers Scheme) support HCC surveillance of all patients at high risk of HCC, thereby reducing the financial concern of most patients. However, our data demonstrated that financial issues are associated with adherence to HCC surveillance. This finding could be explained by the burden of expenses, other than medical expenses, such as travel expenses or loss of a relative’s income because of leaving a job to bring patients to the hospital. 

Recognition of patients at risk of HCC has a major role in adherence to surveillance for HCC. Our results demonstrated an extremely high association between adherence to HCC surveillance and recognizing patients at risk of HCC (OR = 130.396). However, HBV infection was negatively associated with HCC surveillance. HBV infection is the main etiology of HCC globally, especially in Asia and sub-Sahara Africa where HBV infection is endemic. Most patients with chronic HBV infection are asymptomatic, except in the terminal stage. Therefore, patients with undetected cases are unaware of the necessity of HCC surveillance, until symptoms occur. Tumor-related symptoms are usually detected in late-stage HCC and have a variable presentation related to decompensated cirrhosis (Sun and Sarna, 2008). Chonprasertsuk and Vilaichone (2017) demonstrated that HCC patients usually present with an advanced stage of HCC because they were unaware of their risks.

HCC surveillance has been proven to increase early-stage diagnosis and the assessment of curative therapy, and improve survival (Im et al., 2019; Stravitz et al., 2008; Yang et al., 2011). Our results generally agree with these findings, including regular HCC surveillance as an independent preventive factor of detecting a late-stage tumor at the time of diagnosis.

Furthermore, awareness of HBV infection was negatively associated with late-stage detection. The determination of patients’ risk of HCC, thereby resulting in their access to HCC surveillance, has a pivotal role as a preventive factor for late-stage detection. However, awareness of cirrhosis was not associated with the stage of disease detection. This finding could be explained by the proportion of alcohol-related liver disease among cirrhotic patients. Thailand ranks fifth globally for alcohol consumption, and most alcoholic patients continued concurrent alcohol consumption (despite established cirrhosis) and exhibited poor compliance during follow-up. Therefore, alcoholic HCC patients had late disease detection and a worse prognosis than did nonalcoholic HCC patients (Grewal and Viswanathen et al., 2012), which might reduce the effect of awareness of cirrhosis among nonalcoholic patients.

Some limitations of this study should be addressed. First, this cross-sectional study was conducted at a single tertiary care center and included a very small number of participants, only from Thailand. Therefore, the results cannot be generalized. Second, we used the definition of regular HCC surveillance as the assessment of US with and without AFP measurement conducted at least 6–12 months before the first detection of HCC. Surveillance intervals longer than 6 months are associated with noncurative disease (Yeh et al., 2016). This factor may affect the benefits of the surveillance.

A monthly income of >10,000 baths and recognition of patients at risk of HCC were favorable factors. HBV infection was an unfavorable factor for adhering to HCC surveillance. Recognition of HBV infection and regular HCC surveillance were independent preventive factors for the detection of late-stage HCC at the time of diagnosis. We hope that this result will aid physicians in their surveillance of HCC.

## Author Contribution Statement

Conceptualization: AR, AC; Methodology: AR, KA, SC, AC; Formal analysis and investigation AR, AC; Writing - original draft preparation: AR, KA, AC; Resources: AR, KA, SC, AC; critical revisions to the article and Supervision: AC; All authors read and approved the final manuscript.
